# Clinical Audit Assessing Compliance with GP Communication Standards Following Initial Assessments in a Community Mental Health Team

**DOI:** 10.1192/j.eurpsy.2026.11712

**Published:** 2026-07-31

**Authors:** G. Murtaza

**Affiliations:** 1 Child & Adolescent Psychiatry, Children’s Health Ireland; 2 Psychiatry, North Dublin Mental Health Services, Dublin, Ireland

## Abstract

**Introduction:**

Effective communication between community mental health services and general practitioners (GPs) is crucial for maintaining patient safety, ensuring continuity of care, and coordinating follow-up interventions. Both NHS England and the Health Service Executive (HSE) in Ireland provide clear guidance on timely communication following assessments. According to the NHS Standard Contract (2022), outcome letters from outpatient or community assessments should be sent within 10 working days. Similarly, the HSE’s National Clinical Programme for Mental Health (2020) recommends that communication with referring GPs should occur as soon as possible, ideally within 7 to 14 calendar days. Delays in this process can result in fragmented care, increased risk, and avoidable deterioration in mental health conditions.

**Objectives:**

To evaluate whether GP letters following initial mental health assessments were sent within 14 days, in line with HSE/NHS guidelines.

**Methods:**

This retrospective audit included 20 patients who were personally assessed by the auditor at CMHT Swords – Orange Team, North Dublin Mental Health Services, between 13 January and 13 July 2025. Patients included in the audit were those seen for an initial mental health assessment during the specified time frame. Follow-up reviews or emergency consultations where GP communication was not required were excluded. For each case, the audit recorded the date of assessment, date of GP letter dispatch, and the number of calendar days between assessment and communication. Timeliness was categorized into three groups: letters sent within 7 days, between 8–14 days, and beyond 14 days.

**Results:**

A total of 20 patients were included, all of whom had been assessed by the auditor during the audit period. Of these, 15 patients (75%) had GP letters sent within 7 calendar days of assessment, meeting and exceeding the optimal standard. An additional 4 patients (20%) had letters sent between 8 to 14 days, thus remaining compliant with the HSE and NHS benchmark of ≤14 days. Only 1 case (5%) breached the recommended timeframe by exceeding 14 calendar days. Overall, 95% of cases achieved compliance with national communication standards. Notably, 75% of cases were communicated within just one week, demonstrating an efficient and proactive documentation process.

**Image 1:**
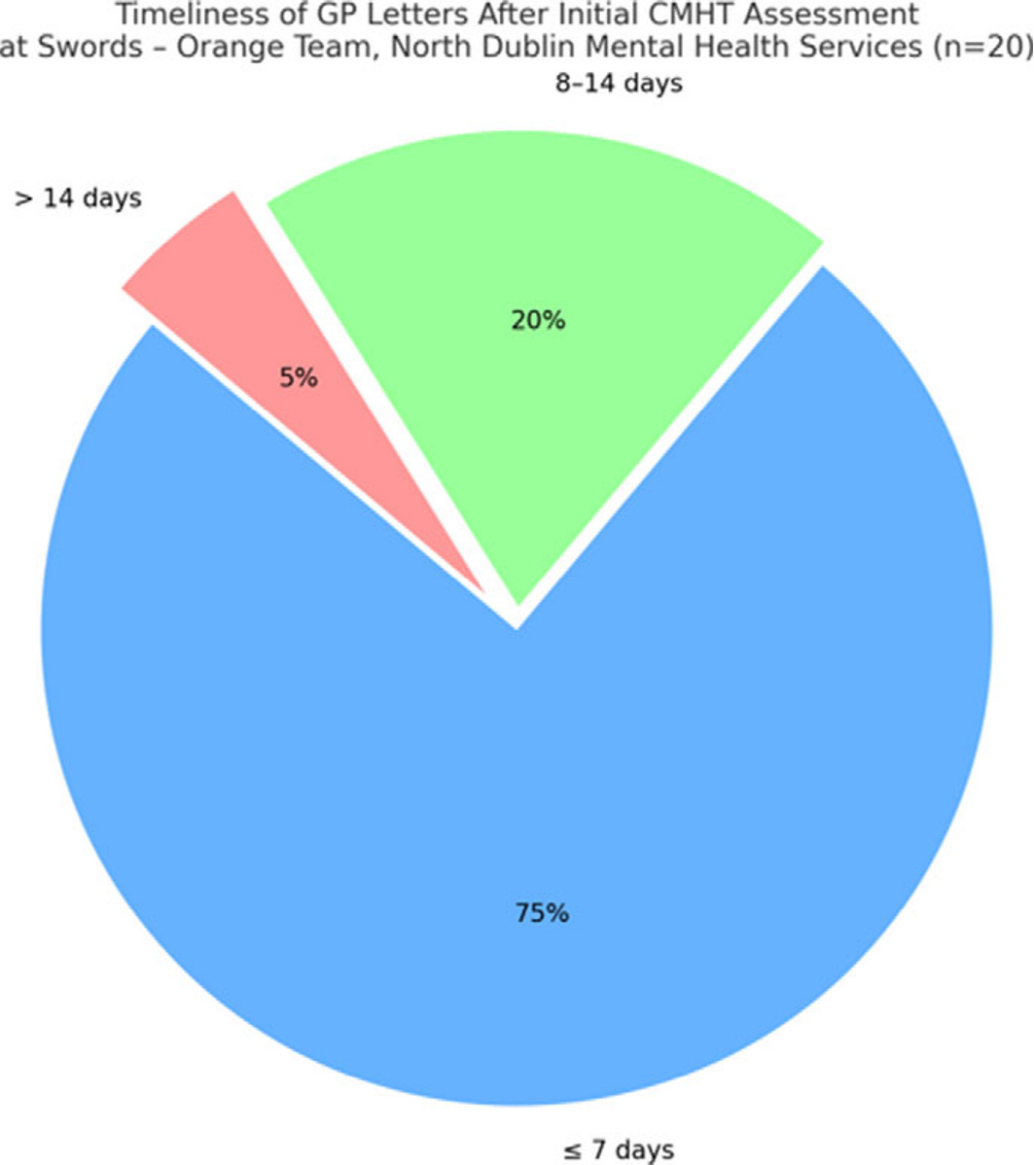

**Image 2:**
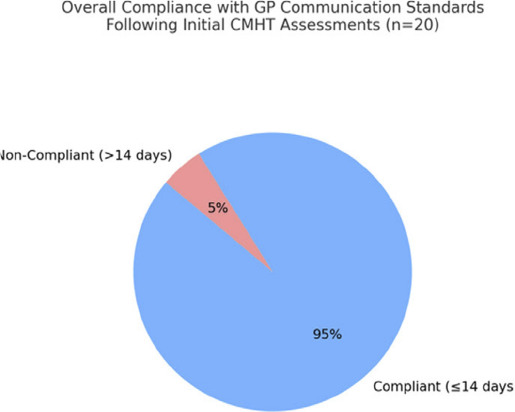

**Conclusions:**

The audit demonstrates high compliance with national standards for timely GP communication following initial CMHT assessments. Continued use of structured templates and weekly review of delayed cases can support full compliance.

**Disclosure of Interest:**

None Declared

